# Hepatitis B surface antigen predicts recurrence after radiofrequency ablation in patients with low hepatitis B virus loads

**DOI:** 10.1097/MD.0000000000009377

**Published:** 2017-12-29

**Authors:** Lan Zhang, Xiao-Ying Xie, Yi Chen, Ning-Ling Ge, Rong-Xin Chen, Yu-Hong Gan, Bo-Heng Zhang, Yan-Hong Wang, Zheng-Gang Ren

**Affiliations:** Liver Cancer Institute, Zhongshan Hospital, Fudan University, Key Laboratory of Carcinogenesis and Cancer Invasion (Fudan University), Ministry of Education, Shanghai, China.

**Keywords:** hepatitis B surface antigen, hepatitis B viral DNA, hepatitis Be antigen, hepatocellular carcinoma, radiofrequency ablation

## Abstract

Radiofrequency ablation (RFA) is a first-line option for the treatment of small liver cancers, but the recurrence remains a problem affecting long-term survival. Hepatitis B virus (HBV) activity is associated with the prognosis of hepatocellular carcinoma (HCC). We investigated the significance of hepatitis B surface antigen (HBsAg) in HCC recurrence after curative RFA treatment in HBV-related small HCC.

We enrolled 404 HBV-related patients with small HCC (≤3 cm) who underwent curative RFA. We used univariate and multivariate analyses to investigate the baseline levels of HBsAg, in addition to other known risk factors for HCC recurrence, for association with HCC tumor recurrence after curative RFA.

The overall 1-, 2-, and 3-year recurrence-free survival (RFS) rates were 75%, 50%, and 34%, respectively. The median recurrence-free time was 25 months. The level of HBsAg was an independent risk factor for recurrence in patients with lower HBV-DNA levels. In hepatitis Be antigen (HBeAg)-negative patients, the 1-, 2-, and 3-year RFS rates were 79%, 64%, and 44%, respectively, for that with low HBsAg levels, compared with 73%, 50%, and 37%, respectively, for that with high HBsAg levels (*P* = .039).

HBsAg might serve as a valuable marker to evaluate the risk of HCC recurrence in HBeAg-negative patients with low HBV viral load.

## 1 Introduction

Hepatocellular carcinoma (HCC) is the fifth most common cancer and the third-leading cause of cancer-related death worldwide.^[1]^ Radiofrequency ablation (RFA) has been accepted as a curative therapy for small cancers of the liver and results in good long-term survival comparable to that of surgical resection.^[2–4]^ The intrahepatic recurrence rate remains high, however, between 28% and 68%.^[5]^ Previous studies have shown that the risk factors for HCC recurrence after RFA include tumor number, tumor size, Child–Pugh grade, alpha-fetoprotein (AFP) level, and serum gamma-glutamyltransferase (GGT) level.^[6–8]^

Hepatitis B virus (HBV) infection is an important etiological factor for HCC in Eastern Asia and sub-Saharan Africa. More than 70% of Chinese patients with HCC have back ground HBV infection.^[9–11]^ HBV viral load has been found to be a risk factor for recurrence after curative resection of HBV-related HCC, so antiviral therapy following resection is encouraged to improve the prognosis.^[12–14]^ The hepatitis B surface antigen (HBsAg) is increasingly recognized as a marker of viral replication and of the response to antiviral therapy in chronic hepatitis B.^[15–17]^ The combination of HBV-DNA level <2000 IU/mL and HBsAg level <1000 IU/mL can be used to identify the carriers of HBV who have minimal risk to develop cirrhosis or HCC. A few studies have shown that high HBsAg levels increase the HCC risk even in patients with a low HBV viral load.^[18,19]^ The significance of HBsAg in recurrence after curative therapy needs to be investigated further, especially in patients with low viral load. We investigated whether a high HBsAg level is associated with increased risk of tumor recurrence in HBeAg-negative patients after radical RFA treatment.

## 2 Patients and methods

### 2.1 Patients

**Figure 1:**
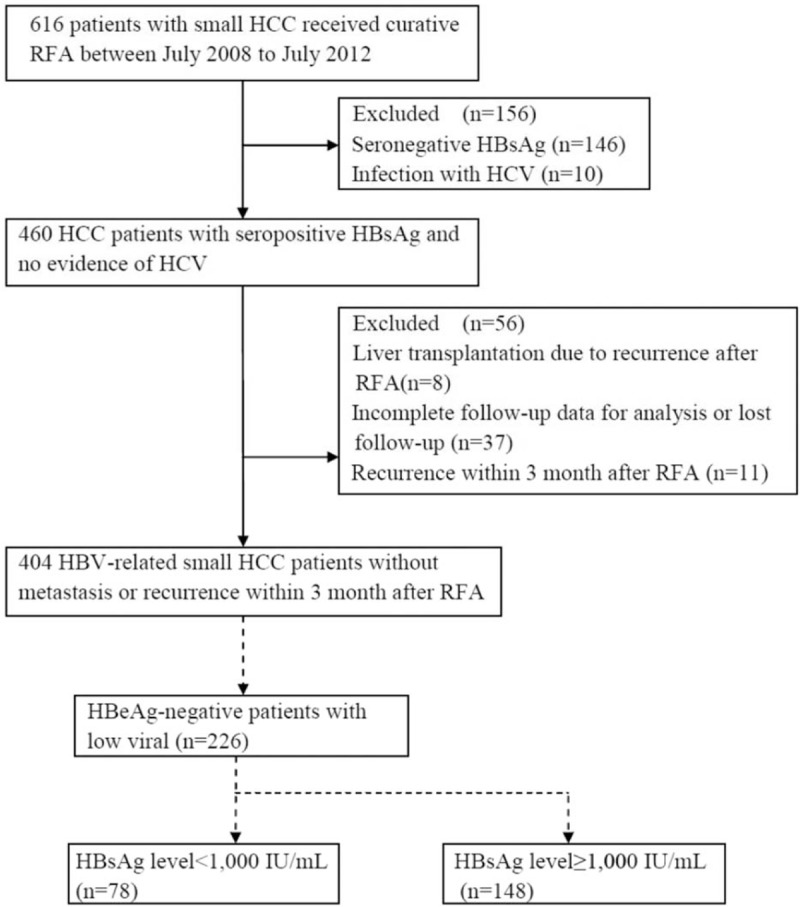
Flow chat of the study for participant enrollment. HBeAg = hepatitis Be antigen, HBsAg = hepatitis B surface antigen, HBV = hepatitis B virus, HCC = hepatocellular carcinoma, RFA = radiofrequency ablation.

The Medical Ethics Committee of Shanghai Zhongshan Hospital provided ethics approval for this retrospective study. We recruited patients from the Liver Cancer Institute, Zhongshan Hospital, Fudan University from July 2008 to July 2012. During that period, 616 patients with small HCC were consecutively treated with RFA. The inclusion criteria for our study were followed as: positive serum HBsAg for at least 1 year; maximal tumor diameter ≤30 mm; tumor number ≤3; Child–Pugh grade A or B; no portal vein invasion. The patients were excluded with: coinfection with hepatitis C virus or human immunodeficiency virus; tumor recurrence within 3 months after RFA; liver transplantation because of recurrence after RFA; or incomplete follow-up data. Thus, we enrolled 404 patients in our retrospective study. Figure 1 shows the flow chart for the patient enrollment.

### 2.2 RFA procedure

The RFA procedure was described previously.^[8,20]^ Briefly, RFA was performed by a percutaneous procedure with conscious sedation and local anesthesia. The patients’ cardiovascular and respiratory systems were monitored during the procedures. RFA was conducted under the guidance of ultrasonography, and overlap ablation was allowed to cover the whole tumor nodule and achieve an adequate safety margin of 0.5 to 1.0 cm when possible.

### 2.3 HBV markers measurement

Serum HBV antigen and antibody were tested using automated chemiluminescent microparticle immunoassay (Abbott Laboratories, Abbott Park, IL). Serum HBV DNA was measured using the COBAS TaqMan HBV quantitative test (Roche Diagnostics, Branchburg, NJ) The HBV serological markers, alpha-fetoprotein (AFP) level, and liver function were measured within a week before RFA.

As patients with low viral loads (HBV-DNA <2000 IU/mL) are usually defined as low-risk HBV carriers.^[21–23]^ An analysis was also performed on HBeAg-negative patients with HBV-DNA <2000 IU/mL, who were divided into HBsAg low and HBsAg high groups.

### 2.4 Follow up and diagnosis of recurrence

The patients were outpatient followed up every 3 months. We measured serum tumor markers and liver and renal function and performed routine blood tests and ultrasonography every 3 months. The patients underwent enhanced magnetic resonance imaging (MRI)/computed tomography (CT) every 6 months. We defined complete ablation as complete devascularization of the lesions during the arterial phase with no appearance of new tumors at other liver sites and incomplete ablation was defined by the presence of an enhancing area on MRI/CT evaluation. If the AFP level was elevated or a suspicious lesion was found, the recurrence need confirmed by MRI/CT based on the American Association for the Study of Liver Diseases HCC diagnosis guidelines. Recurrence was classified as intrahepatic or extrahepatic based on the location and as early or late based on the interval from ablation to the time of recurrence. We defined early recurrence as that occurring within 2 years of RFA and late recurrence as that occurring >2 years after RFA.

### 2.5 Statistical analysis

We expressed quantitative variables as the mean ± standard deviation and qualitative variables as numbers and percentages. Student's *t* tests were used to compare quantitative variables and the χ^2^ test to assess qualitative variables. Overall survival was counted from the time of treatment to the death or date of the last follow up. Disease-free survival was counted by the time from the first RFA to the date of recurrence or the census date of the last follow up. Kaplan–Meier method was used to construct survival curves and the log-rank test to evaluate the difference between groups. The difference was considered as significant when *P* <.05.

## 3 Results

### 3.1 Patient characteristics

The patients’ demographic characteristics are shown in Table 1. The study included 319 men (79.0%) and 85(21.0%) women, with a median age of 55.4 years (range 22–85 years). Three hundred forty-nine patients (86.4%) had a single HCC nodule. Three hundred ninety-one patients (96.8%) had Child–Pugh grade A disease, and 13(3.2%) had Child–Pugh grade B disease. 89 patients had received antiviral therapy before RFA. One hundred eight patients (26.7%) were seropositive for HBeAg. Two hundred ninety-five patients (73.0%) had HBV-DNA level <2000 IU/mL. Two hundred ninety-seven patients (73.5%) had serum HBsAg level ≥1000 IU/mL.

**Table 1:**
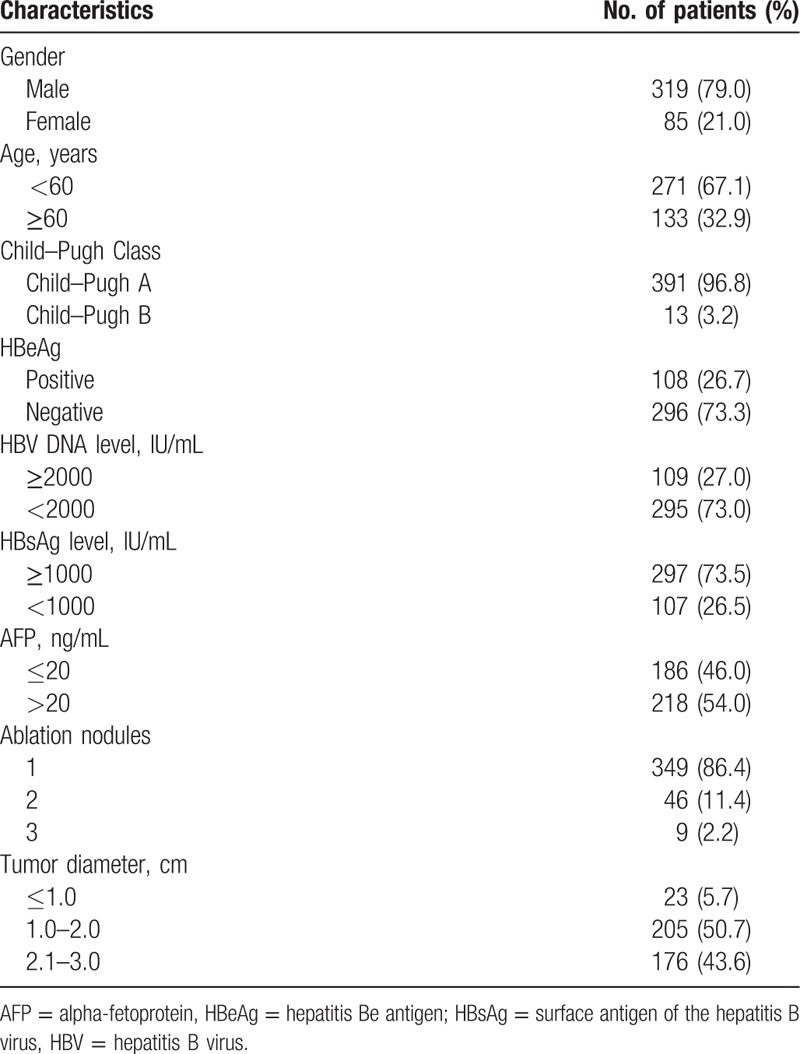
Baseline characteristics of the patients (n = 404).

### 3.2 HCC recurrence after RFA

The median follow-up time was 46.4 months (range 3–98 months). By the end of follow up, there were 285 tumor recurrences (70.5%), of which 279 were intrahepatic and 6 were extrahepatic metastasis. The recurrence was early (within 2 year) in 204 patients and late (after 2 year) 81 patients. The overall 1-, 2-, and 3-year recurrence-free survival (RFS) rates were 75%, 51%, and 34%, respectively, with a median recurrence-free time was 25.0 months.

**Figure 2:**
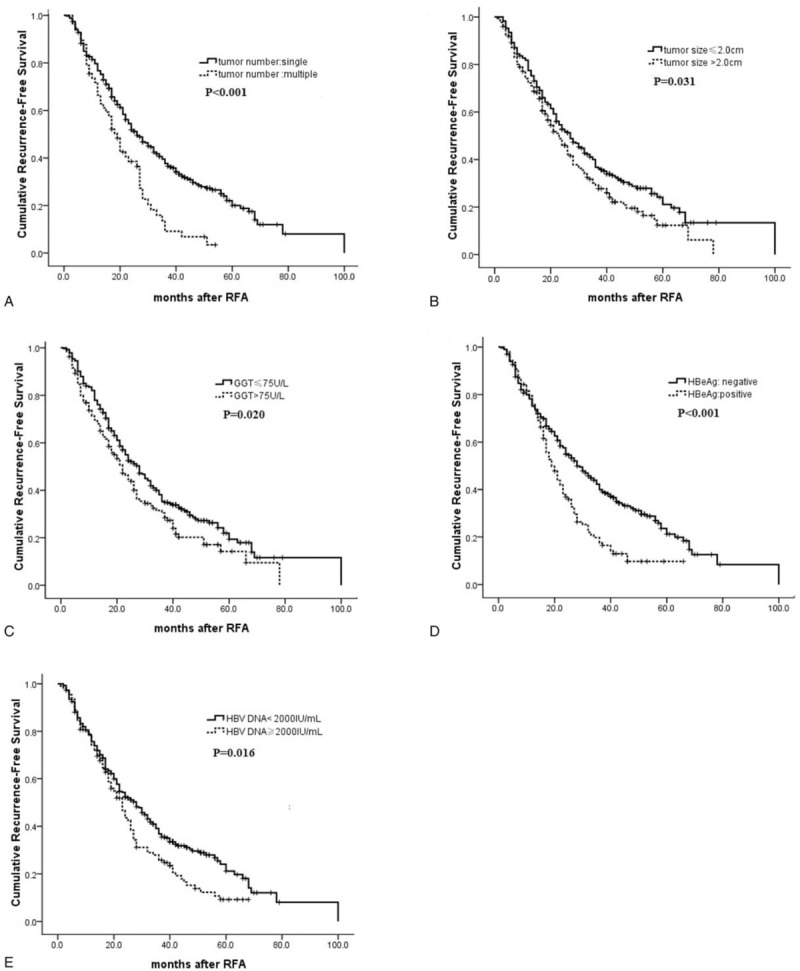
Cumulative RFS according to risk factor. (A) Tumor number (single vs multiple); (B) tumor size (tumor size ≤2.0 vs >2.0 cm); (C) the serum GGT level (GGT ≤75 vs >75 U/L); (D) HBeAg (negative vs positive); (E) preoperative HBV DNA level (HBV DNA ≥2000  vs <2000 IU/mL). GGT = gamma-glutamyltransferase, HBeAg = hepatitis Be antigen, HBV = hepatitis B virus, RFS = recurrence-free survival.

Univariate analyses identified the tumor number (*P* <.001), tumor size (*P* = .031), serumGGT level (*P* = .020), serum HBeAg (*P* <.001), and HBV viral load (*P* = .016) as significant determinants of RFS (Fig. 2). Multivariate analysis identified the tumor number (*P* = .008, HR = 1.580, 95%CI: 1.129–2.212),serum HBeAg (*P* = .024, HR = 1.369, 95%CI: 1.013–1.775), GGT level (*P* = .034, HR = 1.334, 95%CI: 1.022–1.743), and serum HBV viral load (*P* = .042, HR = 1.341, 95%CI: 1.013–1.775) as independent prognostic factors affecting the cumulative incidence of HCC recurrence following curative RFA (Table 2).

**Table 2:**
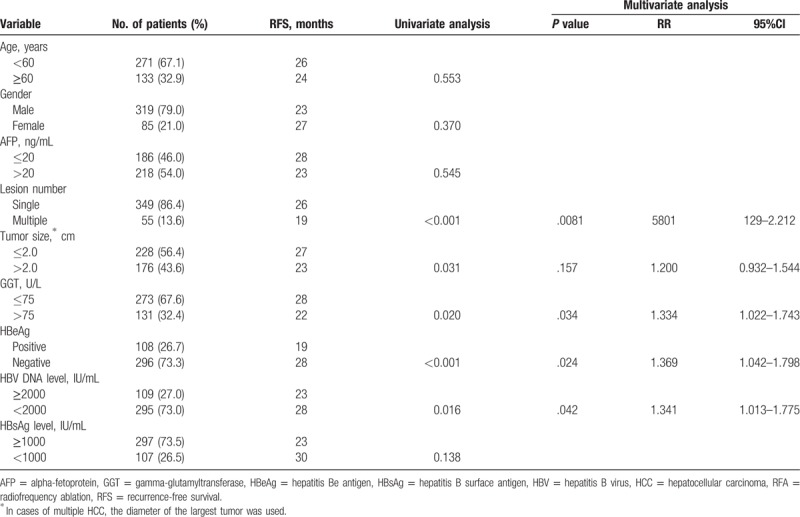
Cox proportional hazards model analysis of predictors of RFS of the patients after complete ablation by RFA (n = 404).

### 3.3 HBsAg level and HCC recurrence after RFA

A recent study showed that high levels of HBsAg increased the risk of HCC in HBeAg-negative patients with low HBV viral load.^[18]^ We therefore investigated the relationship between the serum HBsAg level at the time of RFA and HCC recurrence after RFA in HBeAg-negative patients with low viral load. We performed a subgroup analysis of the 226 HBeAg-negative patients with low viral load (Fig. 1). The correlations between the HBsAg level and clinicopathological characteristics in those patients are shown in Table 3. One hundred forty-eight (65.5%) patients in the subgroup analysis had HBsAg levels ≥1000 IU/mL.

**Table 3:**
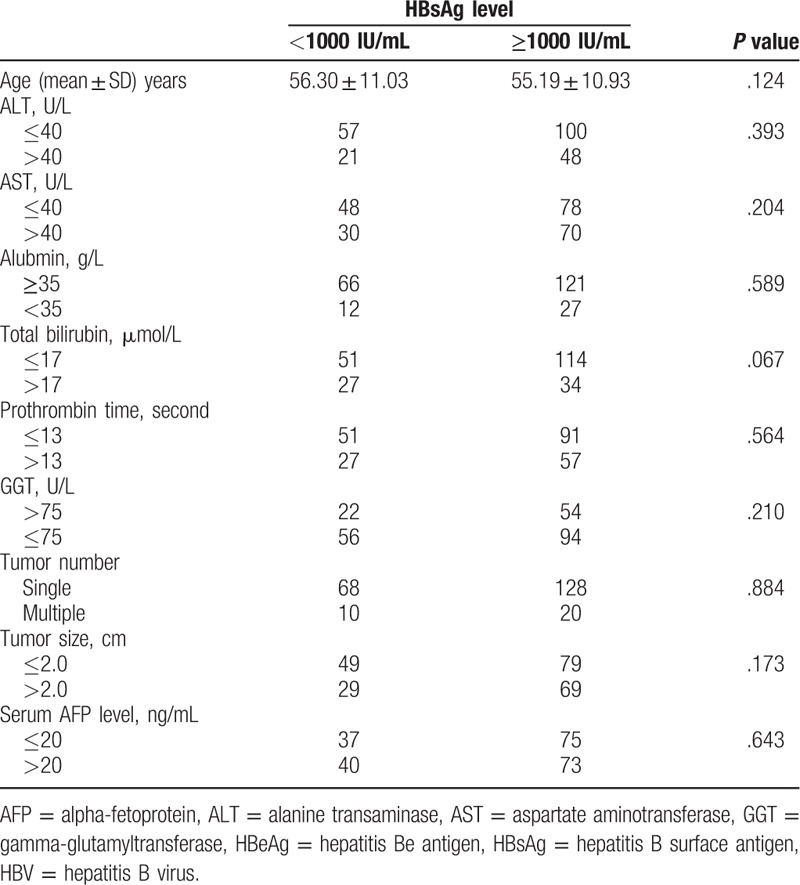
Comparison of clinicopathological features of HCC patients according to the HBsAg level in HBeAg-negative patients with HBV-DNA <2000 IU/mL (n = 226).

The 1-, 2-, and 3-year RFS rates were 79%, 64%, and 44%, respectively among the HBeAg-negative patients with low viral load and high HBsAg levels and73%,50%, and 37%, respectively, among those with low HBsAg levels. The difference between the 2 groups of patients was statistically significant.

In a further analysis of the relationship between the HBsAg level and postablation recurrence, we found that higher serum HBsAg levels had a close relationship with late recurrence (*P* = .038) but not with early recurrence (*P* = .452). We also analysed the level of HBsAg at the time of tumor recurrence. We found there were no significant differences for HBsAg level between at the time of recurrence and at that of baseline.

**Figure 3:**
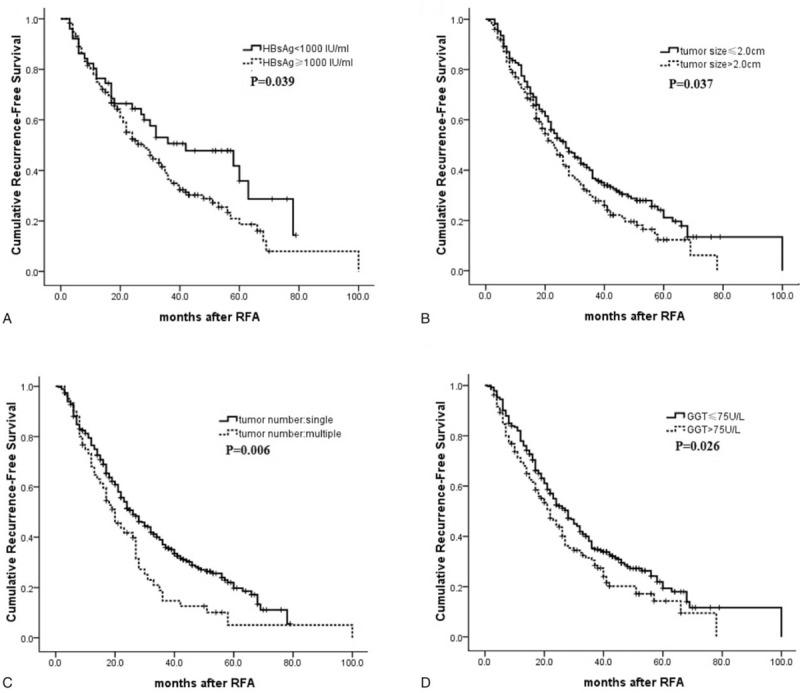
Cumulative RFS according to risk factor in HBeAg-negative patients with HBV DNA level <2000 IU/mL. (A) Preoperative serum HBsAg level (≥1000 vs <1000 IU/mL); (B) tumor size (tumor size ≤2.0 vs >2.0 cm); (C) tumor number (single vs multiple); (D) the serum GGT level (GGT ≤75 vs >75 U/L). GGT = gamma-glutamyltransferase, HBsAg = hepatitis B surface antigen, HBV = hepatitis B virus, RFA = radiofrequency ablation, RFS = recurrence-free survival.

Univariate analyses showed that HCC recurrence after RFA was associated with the HBsAg level ≥1000 IU/mL (*P* = .039), tumor size ≥2 cm (*P* = .037), tumor number (*P* = .006), and GGT level >75U/L (*P* = .026) (Fig. 3). Other characteristics, including age, gender, and AFP level showed no prognostic significance to RFS. Multivariate analysis identified that the tumor number (*P* = .022, HR = 1.684, 95%CI: 1.077–2.633), GGT level (*P* = .038, HR = 1.433, 95%CI: 1.020–2.014), and HBsAg level (*P* = .047, HR = 1.523, 95%CI: 1.059–2.189) were independent prognostic factors affecting the cumulative incidence of HCC recurrence following curative RFA in HBeAg-negative patients with low viral load (Table 4).

**Table 4:**
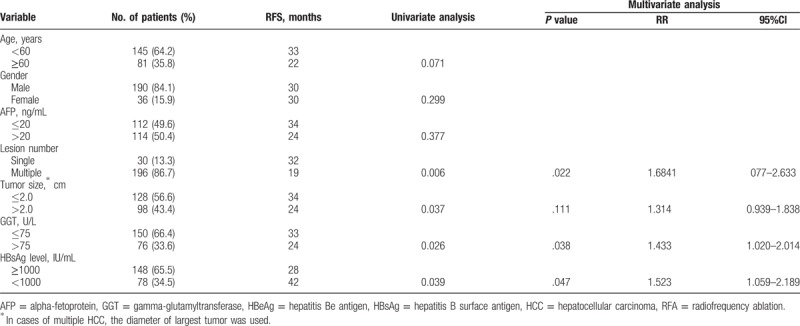
Univariate and multivariate analyses of factors associated with recurrence of the HBeAg-negative patients with low viral load after complete ablation by RFA (n = 226).

## 4 Discussion

In this study, we focused primarily on the correlation between virological factors and the recurrence of small HCC after curative RFA. Our results are consistent with the viewpoint that the HBV viral load is associated with tumor recurrence after curative treatment of HBV-related HCC.^[24–26]^ The risk of HCC recurrence after curative RFA treatment was significantly higher in patients with high viral load than in those with low viral load, which is in accordance with the results that the risk of developing HCC is higher in patients with high HBV DNA and/or HBsAg levels, HBV genotype C>B, and specific mutations.^[27]^ The precise mechanism by which HBV DNA induces recurrent hepatocarcinogenesis is not clearly identified. It is possible that an increased HBV viral load contributes to sustained viremia after the successful treatment of HCC. Active viral replication subsequent to the treatment might increase the likelihood of carcinogenesis in the liver remnant because of the augmented, direct oncogenic potential of HBV and the accompanying inflammatory and fibrotic process resulting from the high viral load.^[28,29]^ In addition, the upregulation of adhesion molecules on the cells lining the sinusoids in patients with active viral replication might enhance the development and spread of tumors.^[30]^ Many studies confirmed that postoperative antiviral therapy reduced late HCC recurrence and significantly improved overall survival among patients with HBV-related HCC.^[31,32]^

We evaluated the role of the HBsAg level in the recurrence of small HCC after RFAin patients with low HBV viral load. An HBsAg level ≥1000 IU/mL was an independent risk factor for HCC recurrence in HBeAg-negative patients with low viral load, and the HBV-DNA level was an independent risk factor for tumor recurrence. Those results suggest that the HBsAg level could be a risk factor instead of the HBV viral load in predicting HCC recurrence, especially in HBeAg-negative patients with a low viral load.

Recently, HBsAg quantification has attracted much attention for its value in stratifying the risk of disease progression and in predicting the treatment response to antiviral therapy in patients with chronic HBV infection. The new 2017 European Association For The Study Of The Liver (EASL) guidelines on HBV Chronic Infection addressed that HBsAg loss, with or without anti-HBs seroconversion, is an optimal endpoint, as it indicates profound suppression of HBV replication and viral protein expression Evidence level II-1 (controlled trials without randomization). Recommendation 1 (strong recommendation)27. HBsAg is a glycosylated envelope protein of the HBV particle, which is produced not only from translated messenger RNAs of transcriptionally active cccDNA but also from integrated HBV-DNA sequences; however, the quantification of serum HBV DNA merely reflects the viral replication activity. Compared with the HBV-DNA level, the HBsAg level provides different but complementary information that might help us to understand patients’ infection status more comprehensively. Serum HBsAg quantification can be useful, particularly in HBeAg-negative chronic HBV infection.^[27]^ There is a positive correlation between the HBsAg and HBV-DNA levels, which is greater at the HBeAg-positive phase, lesser at the HBeAg-negative phase, and least at the low-replicative phase.^[33]^ A recent study showed that patients with low-level viremia who have a high HBsAg level might have an increased risk of HCC.^[34]^ At the beginning, the study demonstrated that both the HBV-DNA level and the HBsAg level correlated positively with HCC development; however, when the study population was limited to HBeAg-negative patients with HBV-DNA levels <2000 IU/mL, in whom the HBV-DNA level has little value in predicting HCC occurrence, the HBsAg level remained the only viral risk factor. Another study showed that HBsAg levels ≥1000 IU/mL were consistently associated with a higher risk of HBeAg-negative hepatitis and cirrhosis. Those findings lend strong support to the hypothesis that a high level of HBsAg increases the risk of HCC in patients with low HBV viral load. A lower HBsAg level can signify adequate host immune control against HBV infection, leading to a decreased risk of HCC. It is of clinical interest to know whether a higher HBsAg level in patients with low HBV viral load would indicate a higher risk of HCC recurrence after RFA. Very recently, Liu et al^[19]^ reported that the HBsAg level was correlated with more aggressive tumor behavior and served as a prognostic indicator in patients with surgically resected HCC with low HBV viral load. Zhou et al^[35]^ further found that in HBeAg-negative patients with low viral load, higher HBsAg levels were associated with late recurrence of HCC but not with early recurrence, whereas HBV-DNA levels were associated with neither early nor late recurrence. Our results demonstrate that HBsAg increases the risk of HCC recurrence following curative RFA. The HBsAg level might serve as a new marker to complement the HBV-DNA level in predicting HCC recurrence in HBeAg-negative patients with low viral load.

Our study was retrospective in nature and used a nonrandomized design. We did not regularly assess the declines in HBV viral load during the follow-up period, nor did we attempt a more detailed investigation of the relationships between HCC recurrence and changes in the viral load after RFA. Further prospective observation should be carried out to identify the significance of HBsAg in HCC recurrence after RFA.

In conclusion, we found that high preoperative viral load (HBV DNA ≥10,000 IU/mL) is associated with worse RFS after curative RFA. In addition, higher HBsAg levels were associated with higher risk of HCC recurrence among HBeAg-negative patients with low viral load.
